# Reviewing the womb

**DOI:** 10.1136/medethics-2020-106160

**Published:** 2020-07-29

**Authors:** Elizabeth Chloe Romanis, Dunja Begović, Margot R Brazier, Alexandra Katherine Mullock

**Affiliations:** Centre for Social Ethics and Policy, Department of Law, University of Manchester, Manchester, UK

**Keywords:** women, interests of woman/fetus/father, reproductive medicine, embryos and fetuses, social control of science/technology

## Abstract

Throughout most of human history women have been defined by their biological role in reproduction, seen first and foremost as gestators, which has led to the reproductive system being subjected to outside interference. The womb was perceived as dangerous and an object which husbands, doctors and the state had a legitimate interest in controlling. In this article, we consider how notions of conflict surrounding the womb have endured over time. We demonstrate how concerns seemingly generated by the invisibility of reproduction and the inaccessibility of the womb have translated into similar arguments for controlling women, as technology increases the accessibility of the female body and the womb. Developments in reproductive medicine, from in vitro fertilisation (IVF) to surrogacy, have enabled women and men who would otherwise have been childless to become parents. Uterus transplants and ‘artificial wombs’ could provide additional alternatives to natural gestation. An era of ‘womb technology’ dawns. Some argue that such technology providing an alternative to ‘natural’ gestation could be a source of liberation for female persons because reproduction will no longer be something necessarily confined to the female body. ‘Womb technology’, however, also has the potential to exacerbate the labelling of the female body as a source of danger and an ‘imperfect’ site of gestation, thus replaying rudimentary and regressive arguments about controlling female behaviour. We argue that pernicious narratives about control, conflict and the womb must be addressed in the face of these technological developments.

‘As all historians know, the past is a great darkness, and filled with echoes.’

― Margaret Atwood, The Handmaid's Tale

## Introduction

This article traces how attitudes to female reproduction, shaped by historical misunderstandings of procreation and the female body, have perpetuated an approach that continues to subjugate and ‘other’ women,^1^ especially as they gestate and bear children. From classical times, the womb garnered suspicion and fear among ‘medical men’, theologians and ordinary people partly because it was obscured from their view. Pernicious narratives about conflict and danger, born from ignorance, have endured and transmuted into modern, medicalised tropes. New reproductive technologies, heralded as increasing reproductive choice for women, equally foreshadow exacerbation of maternal–fetal conflict and medical hegemony over women’s choices. We illuminate this problem and argue that such attitudes must not be permitted to direct ethico-legal approaches to emerging technology.

Until recently, the womb was an exclusively natural, static female organ, but medical science is now delivering opportunities to transplant^2^ or emulate the function of the womb.^3^ Women who suffer from uterine factor infertility can now receive a uterus transplant, and it seems feasible that soon trans women and cis men wanting to gestate their own child could too.^4^ Such advances may potentially degender^5^ gestation. On the horizon, there is the promise of ‘artificial wombs’^6^ creating further options for the wombless and those who want a child but not to gestate. More choices for all putative parents and better healthcare for the fetus, whether in a parent’s biological uterus or a ‘machine,’ appear to represent progress which should be welcomed.

History, however, suggests that a note of caution must be voiced about the impact of such developments on women’s rights and role in society more generally. Fascination with the womb, coupled with the capacity for others to intervene for the benefit of the fetus, has culminated in the notion of ‘maternal–fetal’ conflict, in which the interests of pregnant woman and the fetus are presented as incompatible with, or in competition with, each other. While advances in reproductive technologies offer hope and important solutions for putative parents, there may be unintended side effects that negatively affect pregnant women because of prevailing narratives within healthcare, maternity care and wider society.

In this paper, we recall how frameworks based on the womb as a site of conflict, and concerns about the need to control women because of their wombs, are evident in medical practice and law throughout history. We then consider how these narratives have prevailed as advances in medical technologies have provided us with a ‘view into the womb’, and demonstrate why the conflict framework is not only conceptually and evidentially unjustified, but also potentially harmful. Finally, we examine this danger in connection with future reproductive technology, focusing on ‘artificial womb’ technology, to interrogate these issues in a contemporary context. We consider the development of ectogestation and argue that such technology exemplifies further why we need a wholescale shift in medicine, ethics and law away from narratives that consider pregnancy and the womb as a site of danger.

## Hidden from view

Possession of a womb has not always been a blessing. In the past, the woman who successfully gestated children faced an agonising labour and risk of death. Her pain was to expiate the sin of Eve in tempting Adam with that apple. The book of Genesis declares ‘I will greatly multiply your pain in childbirth, in pain shall you bring forth children yet your desire shall be for your husband’ (Genesis 3.7). John McKeown cites Martin Luther:

[W]e see how weak and sickly barren women are. And those who are fruitful are healthier cleanlier and happier and even if they bear themselves weary—or ultimately bear themselves out— that does not hurt let them bear themselves out this is the purpose for which they exist.^1^

For Luther, women were ‘not created for any other purpose than to serve man and be his assistant in bearing children.’^1^

However, even if a woman’s purpose was thought to be to gestate her husband’s children, her contribution to the creation of the child was judged by many learnt men across the ages to be simply a ‘seed bed’ for the embryo.^2^ Wombs were no more than a necessary medium in which the father’s seed could grow. Aristotle argued that the embryo was formed when the male seed interacted with menstrual blood. The woman nourished the seed.^2^ Galen disagreed, contending that women produced seeds, although ‘weaker in nature’ than the male seed. In the 17th century, anatomists examining semen under the microscope discovered sperm, originally described as ‘animalcules.’ The view (described as preformationism) grew that the fully formed child was present in the sperm.

Animalculism obviously proved inaccurate, but for those who believed that mothers only contributed an environment in which the father’s child could grow and be nurtured, the woman was in effect a ‘gestational carrier’. From this (mistaken) premise, the legal incapacities which English law imposed on married women begin to make some sort of sense.^3^ The marriage contract obliged a wife to make her womb available to nourish her husband’s s children. Coupled with the myth that a wife could not refuse consent to marital intercourse, perpetuated in English law until 1991,^4^ husbands enjoyed something akin to what we might classify today as a right to procreate, and wives a duty to provide the means by which he might do so.^7^

A husband’s interest in the child was magnified by the firm belief that the child was ‘his’, the product of his body; he had the strongest of interests in ensuring that no other man’s ‘animalcule’ was carried in the wife’s womb and passed off as his. He had a further strong interest in ensuring that the behaviour of the ‘gestational carrier’ did not compromise his reproductive enterprise. That sadly did not mean that all husbands acted positively to promote the health of the wife. The high rates of child mortality and at many times in history the surplus of women over men might mean that quantity in reproduction was the primary objective, to generate as many children as possible and replace ‘worn out wombs’ with fresh stock. The desire for sons and primogeniture begin to make sense. If you accepted animalculism, a son when he reproduced begat a grandson who shared your blood. Daughters will bear a child formed by her husband’s ‘animalcule’, unrelated to its maternal grandfather.

A working womb did not necessarily benefit the woman, but to be barren might have been a worse fate. From classical times, theologians and physicians declared barren women to be monstrous. In Ancient Greek myth, the grisly Gorgon queen Medusa, whose gaze turned men to stone, was said by some to be barren. The empty womb was dangerous, but so was any womb, dangerous to the woman and to others. Secreted far from public view, wombs were judged the cause of many female ills, or rather conditions styled ‘ills’ by men.

Women, learnt men declared, were defective creatures possessing weak intellectual capacity and unregulated emotions. Christian theology was supported by so-called science. The ‘scientific’ grounds for female defects were various, contradictory and changed over time. When it came to female physiology ‘medical men’, anatomists, the law, the Church *et al* resembled Alice in Wonderland trying ‘to believe as many as six impossible things before breakfast.’ The female body was declared to be defective compared with male perfection, yet when anatomists were able to examine the interior of female corpses, they argued that female organs could be seen as inversion of the male.^5^ So, it was said that the ‘neck of uterus is like the penis, and its receptacle with testicles and vessels is like the scrotum’.^2^ Wombs, however, were accorded dark powers not shared by the perfect male genitalia. The ‘wandering womb’ which was not fixed in its proper place but wandered around the body pressing on heart and lungs endangered the woman’s life resulting in ‘suffocation of the mother’.^6^ The wandering womb was described as ‘a migratory uterus prowling about the body like a wild animal pressing on the chest.^7^ The uterus emitted noxious fumes; not a desirable commodity. By no means all eminent physicians agreed that such a condition existed. The Trotula, a medieval compendium on women’s medicine, rejected the notion of ‘suffocation of the mother’.^8^ Popular opinion on science then as now influenced society, as Edward Shorter explained ‘through popular culture as well rode a visceral male fear of women’s’ magical powers’.^9^ Wandering wombs made a good story. A cure for wandering wombs and later hysteria recommended by some medical men was sexual intercourse—within marriage of course. Writers warned of the libidinous nature of imperfect women, seeking in sexual relations with a man to be completed. As Rawcliffe notes, male writers seemed to see no contradiction in depicting the womb as both ‘a passive empty vessel and a voracious animal’.^7^

If the danger of the womb was not enough, its monthly function testified further to the evidence of female defect. Menstruating women were ‘venomous during the time of their flowers and so dangerous that they poison beasts with their glance and little children in their cots.’ Should a man have intercourse with a menstruating woman, a child conceived might interalia be born leprous or blind, hunch backed or malformed.’ Any child born in defiance of such a taboo ‘would bear some mark of ignominy, if only red hair’.^7^

Once human dissection showed plainly that wombs were not apt literally to suffocate or wander around the body, Victorian doctors recast the womb as the cause of hysteria.^10^ The womb disordered the female brain. We hear a great deal about ‘baby brain’ today and cognitive impairment in the menopause. It has been reported that women having a heart attack with exactly the same symptoms as men are often sent home told they are suffering from panic or stress^11^—hysteria by any other name?

Arcane beliefs about the womb, which underpinned laws adverse to women and especially pregnant women, no longer hold sway. The womb is no longer mysterious and yet misogynistic attitudes, which define women by their biology, persist. Look at contemporary social media abuse of female MPs. See how some US states have passed regressive laws on abortion, contra to Constitutional Rights, to police every woman’s womb.^8^ As we now examine, technological advances, while potentially benefiting women, might also invite opportunities to interfere with female autonomy, increasing the potential for conflict between the interests of women and fetal welfare and the continued pathologisation of aspects of female physiology.

## A view into the womb

The previous section outlined the ways in which the ‘inaccessibility’ of the womb was a source of rampant speculation about women and their pregnancies. Twentieth century advances in medical technology have drastically changed how we engage with women and the fetus during pregnancy, though as we will demonstrate, these have not necessarily quashed some of the backward thinking about needing to control gestation. X-ray technology initially allowed obstetricians to diagnose potential health problems prenatally, and the later development of obstetric ultrasound provided a safer way of gaining insight into fetal health, ultimately becoming a routine part of prenatal care. In the second half of the century, various forms of prenatal testing and treatment procedures were pioneered, including complex prenatal surgeries for conditions like spina bifida.^12^ Many of these relatively recent developments are now used routinely, and some previously experimental and risky procedures have been made safer and less invasive, allowing their gradual introduction into healthcare.^13^ These developments have placed the fetus firmly at the centre of the gestation process. Some worry that this shifts the maternal–fetal relationship to be potentially adversarial and may also lead to the woman’s interests being side lined.^14^ Douglas explains that ‘the perception of childbearing as primarily, rather than coincidentally, a health matter has led to an increasingly more difficult dilemma for health professionals. Who is their patient, the mother or the fetus?… But in the event of a conflict of interests who should take priority?’.^15^ Technology has arguably oriented the focus away from the pregnant person towards gathering as much information as possible about the fetus, potentially becoming a form of coercive control. Douglas concludes that ‘the main focus of attention these days [with all of contemporary obstetric technology] has moved away from the pregnant women and towards the fetus within her… [and this] enables supervision to be maintained over the woman and to some extent her lifestyle’.^15^

Developments in fetal medicine, from heart rate monitoring to three-dimensional imaging and prenatal surgery, have made the journey from zygote to child, once hidden from view, accessible not only to pregnant women, but also their families, doctors and society. We are increasingly afforded a ‘view into the womb,’ leading to the perception of the fetus as a distinct being. Taylor notes that ultrasound has had the effect of bringing fetuses ‘to life’ in that ‘it necessarily involves making visible the invisible and unmasking what has been hidden and obscured, [and] inevitably draws us into a rhetoric and politics of vision.’^16^ The fetus appears as something that can be watched and ‘interacted with’.^17^ Technology has afforded the means ‘to monitor, to control and possibly intervene.’^18^

However, an important boundary remains in the form of the pregnant woman, whose consent is essential for any kind of intervention to be performed: ‘literally, if not conceptually, the pregnant woman incorporates the foetus, so direct medical access to the fetal patient is as remote as ever’.^19^ Laws in many countries appear to recognise the interests of the pregnant woman as primary, and the fetus is usually not considered a being with its own rights and interests.^20^ Respecting the autonomy of the pregnant patient is given ethical primacy even by those who would accept a limited notion of fetal patienthood.^21^ Yet it is necessary to be vigilant as personal and social perceptions of fetal status and interests have and are likely to continue to evolve, even as legal and ethical codes maintain the autonomy of the pregnant woman as central.^22^ The medicalisation of pregnancy has already led to a change in how women perceive their responsibilities to the unborn child,^23 24^ and technological developments, such as more sophisticated prenatal imaging or pregnancy apps monitoring fetal well-being, could further encourage this thinking. Empirical studies of pregnant women preparing for prenatal therapy suggest that the fetus is commonly seen by them as a distinct entity with its own needs and interests.^25^ Further technological development may increase the potential for tension between the perceived interests of the woman and her fetus. Consequently, it is important to interrogate the ways in which we imagine the maternal–fetal relationship as technology increases access to the womb.

There is an urgent need to avoid perceptions of the womb as a site of conflict, in order to ensure that pregnant women’s bodies are not treated as a dangerous environment for the fetus, rather than an essential part of the maternal–fetal unit. Pregnant women’s interests and autonomous choices must not be erased and ignored in favour of promoting fetal well-being, and the conflict view of the maternal–fetal unit seems to play a crucial role in this framing. In the next two sections, we present the notion of maternal–fetal conflict as it is often used in the ethical and legal literature, and demonstrate why this notion is unsubstantiated, incoherent and possibly dangerous, and should therefore be rejected.

## Reviewing conflict

Maternal–fetal conflict is said to occur when a pregnant woman behaves in ways that may be harmful to the fetus, such as drinking excessive alcohol or refusing a caesarean that is medically indicated.^26 27^ This is seen in definitions like: ‘maternal–fetal conflict has been defined as the situation in which the intent or actions of the pregnant woman do not coincide with the needs, interests, or rights of her fetus as perceived by her obstetric caregivers’.^28^ This posits the main ethical dilemma for doctors as being how to balance the interests of the pregnant woman in having her autonomy respected and the fetus in having its ‘interests’ or welfare protected, leading back to the problematic issue of recognising the fetus as a separate patient.

Sometimes it is not the pregnant woman who is considered to be the ‘perpetrator’ of the conflict—her well-being might be jeopardised by interventions aimed at ensuring the well-being of the fetus^29^—for example, more invasive maternal–fetal surgeries that may present long-term risks to the woman’s health and well-being, and sometimes caesareans performed for fetal benefit. The definition above more clearly paints the pregnant woman and fetus as adversaries, rather than acknowledging that the well-being of the fetus ultimately depends on respecting the autonomy of the pregnant woman, who is usually the one most invested in ensuring good outcomes for the future child.^30 31^ However, any conception of clashing interests rests on the assumption that pregnancy involves two separate parties, between whom conflict might occur, rather than a necessarily interdependent biological unit. We argue instead that this interdependence must be taken as a starting point when examining ethical issues in prenatal care and application of reproductive technology.

The notion of ‘maternal–fetal conflict’ is so pervasive it is often the starting point of discussions related to ethical issues in pregnancy. Bioethical discussion often takes this framework, and examples of conflict, as the default assumption^32 33^ or a problem to be addressed,^34^ thus generating the false perception that such conflict is widespread. Medical research also adopts this terminology at times, which inevitably frames the presentation and discussion of findings.^35 36^ Most notably, maternal–fetal conflict is arguably one of the key concepts in the area of obstetric ethics,^21 37 38^ including a large body of work on balancing the doctor’s obligations towards the pregnant woman and those owed to the fetus.^39–41^ Some have suggested that the difference of opinion between medical professionals and pregnant woman about what to do in a particular situation is the true source of conflict: the term ‘maternal–fetal conflict’ ‘misdirects attention away from the conflict that needs to be addressed: namely the conflict between the pregnant woman and others (such as child welfare agencies, physicians and other healthcare providers) who believe they know best how to protect the fetus’.^26 42^ This is reminiscent of the imagery conjured by pre-Victorian doctors treating the female body as an innate source of danger. We can see echoes of suspicion and mistrust towards women where risk is calculated by doctors who seem to be advocating for the fetus, as if the default assumption is that women’s behaviour will somehow endanger it.

### Conflict enshrined in the law: the example of England and Wales

The law is often the mechanism through which ethical and medical ideas about conflict in pregnancy have been translated into a substantial impact on women’s bodies and choices. McLean explains that ‘the attribution of rights to embryos and fetuses places the mother and conceptus in direct conflict in a number of possible situations’.^43^ There are several legal principles which afford recognition to fetuses in ways influenced by conflict framing. Alghrani notes that ‘many of the cases that have generated legal rules and principles on the status of the unborn have developed in the context of the abortion debate and cases of maternal–fetal conflict’^44^ Thus, they have some notion of inherent conflict at their root.

In England and Wales, it has been established by the courts that the fetus does not have legal personality until birth, and therefore, it does not (and probably never did) have any claim to human rights protection.^45^ Moreover, an unborn child cannot be the victim of murder, and manslaughter may only apply if it is delivered alive before subsequently succumbing to its injuries.^9^ ^46^ The fetus cannot be a victim of a non-fatal offence against the person irrespective of whether it survives the injury.^47^ A fetus can be, however, the victim of child destruction once it has reached the gestational stage of being capable of life outside its mother’s body.^48^ The offence of procuring a miscarriage also safeguards fetal life unless one of the grounds specified in the Abortion Act 1967 applied.^49^ ^10^ While the case of Paton,^45^ which involved an unsuccessful claim by a putative father seeking to prevent abortion, confirmed that the fetus has no right to life under Article 2 of the European Convention on Human Rights,^11^ abortion law does provide certain protections for fetal life. Section 1 (1)(a) Abortion Act 1967 can be seen to provide little, if any, protection for fetal interests up to 24 weeks gestation, but it is possible for doctors—as gate-keepers—to exercise professional discretion in seeking to discourage abortion, or indeed to refuse to participate as a matter of conscience.^46^ It also might be argued that the first ground in the Abortion Act provides real protection to a non-viable fetus because it requires women to justify their terminations in medical terms (though in reference to their own bodies). It remains unlawful for a pregnant person in English law to access termination ‘for any reason or no reason’.^51^ After 24 weeks the potential for maternal–fetal conflict within the Abortion Act 1967 is more significant. We see, therefore, that abortion law and the Infant Life Preservation Act 1929, in offering greater protection once there is the potential for the fetus to survive ex utero, convey the message that the mature fetus has interests worthy of protection.

For women who have chosen to carry a pregnancy to term, other points of conflict arise. The shift towards greater respect for patient autonomy in medical matters has been slow to materialise in disputes involving pregnant women. The forced caesarean cases illustrate this problem.^52–54^ Although the rights of pregnant women to refuse interventions intended to benefit their fetus are routinely declared in judgements,^52 53^ implementation of these principles is hard to evidence since the majority of these cases involve compulsory treatment being ordered on the grounds that the woman does not have capacity. Some of the ways in which women are found to be lacking in decision-making capacity are questionable.^12^ Conversely, professional reluctance to allow women to choose to give birth by caesarean, illustrated in Montgomery v Lanarkshire,^55 56^ suggests that the autonomy of pregnant women is often not prioritised. Women seeking to avoid medical interference in childbirth altogether will also find their choices constrained. Article 45 of the Nursing and Midwifery Order 2001 makes it a summary offence for a person other than a registered midwife or medical practitioner to attend a woman in childbirth, unless there is sudden or urgent necessity. This is a formalised attempt to medicalise pregnancy and childbirth and take away control from the labouring woman. As such it is reminiscent of the medical comment repeatedly made of female physiology throughout history. Such instances of conflict in childbirth seem to support the view that the true conflict lies between women and the medical profession,^26^ and that the presence of the fetus still means that a woman is less likely to be afforded full agency in situations where her views conflict with accepted ideals about what is ‘best for baby’. Even more extreme examples are found in the USA where a pregnant woman’s status as an aggressor is embedded in a wide range of criminal laws including the Federal Partial-Birth Abortion ban and fetal homicide laws at State level.^57^

## Reframing conflict

The prevalent framing of pregnancy as a site of conflict in medicine, ethics and law has been challenged, especially by authors writing from a feminist standpoint. Bowden argues that the pervasive maternal–fetal conflict conceptualisation of pregnancy is both innately problematic and empirically unfounded.^30^ She explains that this model ‘presents the interests of the pregnant woman as conflicting with those of the future child and therefore, the pregnant woman as a threat to her future child rather than the person who is most invested in its welfare’.^30^ This can lead to the ignoring of women’s autonomous choices as well as the erosion of trust between pregnant women and medical professionals, likely leading to further negative outcomes. In this section, we demonstrate that there are conceptual, outcome-based, and political and social reasons why framing pregnancy as a site of conflict is both unfounded and harmful, and must be abandoned.

First, the notion of maternal–fetal conflict is arguably conceptually unsound. This has been explored extensively within bioethical and philosophical literature. There are metaphysical arguments about the status of the pregnant woman positing that it is mistaken to consider a pregnancy as involving two distinct entities.^58 59^ Some argue that considering the fetus as a part of the pregnant woman^59^ or considering the fetus-pregnant woman as a unit/dyad view^19^ is more accurate. Some of these authors do not attempt to draw any normative claims from such argumentation.^59^ Still, their conclusions could be used as support for the idea that the pregnant woman and the fetus are intertwined such that the concept of there being separate interests cannot make sense.

The terminology around this concept is also highly suggestive and value-laden. Using the term ‘conflict’ perpetuates the problematic assumption that ethical dilemmas in pregnancy are a matter of clashing rights between the woman and the fetus,^26^ when it is not determined in either ethics or law that fetal rights are a coherent concept.^20 60^ Also, the ‘maternal’ in maternal–fetal conflict implies that the pregnant woman already has parental responsibilities towards the fetus while it is still in the womb, which may then conflict with her other desires and actions. This is also (rightfully) contested,^26^ with some authors arguing that fetuses cannot be the proper object of parental responsibilities.^61^

Second, the outcomes for maternal and fetal health are worse when women are perceived as a potential threat to their own pregnancy. As the fetus is increasingly visualised and subject to clinical recognition as a ‘patient’, and even some legal recognition,^13^ this strengthens the perception that there is a need to interfere with the choices women can make about their pregnancies, either by failing to disclose information (as in Montgomery^55^) or in the framing of childbirth as an emergency when this may not necessarily be appropriate.^62^ However, empirical studies have demonstrated that fetal outcomes are better when women are enabled to take a more directive role in their own care.^30 63 64^ Respecting women’s autonomy is important in allowing them, the people most familiar with their own body, underlying health needs and values, to make the decisions they feel best promote their own and their fetus’s welfare.

The notion of conflict is deeply rooted in a historical tradition of thinking about women and wombs. The origins of our social and medical attitudes can be found in early mistaken beliefs about procreation and the mother’s gestational role. These ideas however, when applied in medical practice, encourage dysfunctional relationships between clinicians and pregnant women, as observed in forced caesarean cases where doctors often seek court approval in cases involving women with mental health conditions.^65^ The presentation of a woman’s health interests and personal well-being as detrimental to her fetus can also dissuade some, particularly vulnerable women, from accessing prenatal care.^30 66^ Pregnant women are more likely to engage in prenatal care when they do not fear legal consequences^67^ or being made to feel judged by care providers.^30^ There is substantial evidence that outcomes are better for both woman and fetus when pregnant women are engaged and receive routine prenatal care,^68 69^ so to guarantee this autonomy in pregnancy must be protected. Furthermore, as Bowden observes, women choosing pregnancy are almost always invested in the outcome and so treating women as a source of danger is usually spurious.^30^

Finally, there are significant political and social ramifications of the framing of the womb as a hostile environment. Some of these are already evident in practice. A worrying trend of prosecuting ‘pregnancy-related offences’ in some US states under so-called ‘fetal protection laws’ shows a perception of women as dangerous, leading to apprehension and all the consequences of life after imprisonment. These cases involve an over-representation of poor women/women of colour, showing how certain groups are disproportionately affected by conflict framing, depending on the overall political context.^70^ Such thinking also encourages the view of women as ‘dangerous creatures’ that threaten a man’s procreative interests, again echoing the themes evident in the historical background provided earlier in this paper. Furthermore, we have demonstrated that it is not constructive, nor pertinent to the achievement of the best clinical outcomes, to routinely place blame at women’s feet for failing in gestation when there are other factors that need to be addressed. There are broader socioeconomic factors that are more responsible for poor prenatal outcomes, including access to care, than any individual women’s behaviour.

We argue that the above considerations show we must ‘move away from presenting the needs of a developing fetus as being in conflict with those of the pregnant woman’.^30^ One way to do this is by adopting a more holistic view, which regard the pregnant woman and the fetus as ‘an inseparable whole whose well-being needs to be fostered before, during and after the pregnancy’.^28^ Focusing on this maternal–fetal ‘dyad’^19^ as an interdependent biological unit is a better approach to providing ethical prenatal care than trying to balance the distinct interests of two (seemingly opposed) parties, especially since the fetus is fully dependent on the pregnant woman for its health and survival.^19^ This also ensures that women are affirmed as persons, with their autonomy and bodily integrity respected. Rejecting the notion of ‘conflict’ reduces the risk of stigmatising pregnant women for a multitude of decisions about their gestation, from diet to childbirth. As future reproductive technologies emerge, it is particularly important that we reframe thinking about pregnancy to determine appropriate ethical and legal parameters for their use.

## Womb with a view

One of the most anticipated developments in assisted reproduction is ‘assisted gestation’; the ‘artificial womb.’ Ectogestation^58^ is the process of gestation undertaken ex utero in a device attempting to emulate the conditions of the human womb. Complete ectogestation is the growing of babies entirely from scratch in an artificial womb; partial ectogestation is the use of ‘artificial womb’ devices to facilitate the continued gestation of human entities that are removed from a woman’s womb prematurely. Recent animal experiments with artificial womb prototypes have demonstrated it is possible to facilitate partial ectogestation in lambs,^71–73^ fuelling speculation about the development of this technology and its impact.

Artificial wombs are often heralded as a source of potential liberation for women. Simonstein and Mashiach-Eizenberg explain that ‘reproductive hazards have traditionally been viewed as women’s fate, and therefore, have been taken for granted’.^74^ Firestone,^75^ Kendal^76^ and Smajdor^77^ echo concerns about the physical burdens of gestation and pregnancy being placed exclusively on female people and posit that entirely removing gestation from the body offers women, finally, equal opportunity. Smajdor explains that with complete ectogestation available, women would be able to ‘reproduce as men do, without risking their physical and mental health, economic and social well-being, and crucially—their bodily integrity’.^77^ Partial ectogestation has also been advocated as beneficial for women as a way of alleviating some of the burdens of pregnancy by offering, for example, an alternative if pregnancy is dangerous (or potentially undesirable) in the later stages.^78^ The problem with the arguments about how ectogestation might assist women in taking more control of their reproduction is that they are often advanced in a vacuum, seemingly ignorant of contemporary sociolegal conditions and importantly, women’s histories. Some of our concerns about the capacity of the technology to liberate women of the burdens placed exclusively on the female body are shared by other feminist scholars.^79–81^ Vallerdu and Boix assert that ‘medical practices have historically maintained a form of male control over women, and that reproductive technologies have been oriented towards the male help in detriment of women’s welfare’^82^ and thus the introduction of ectogestation would likely be no different.

In this section, we place the (potential) development of the artificial womb into historical and contemporary context by demonstrating how prevailing narratives of maternal–fetal conflict—if not addressed—will limit the capacity of technology capable of ectogestation from benefiting women and pregnant people. First, artificial wombs might escalate the pathologisation of gestation, and second, they might fuel excessive control over natural pregnancy by creating a ‘narrative of alternative.’ The purpose of this examination is not to advocate that we should ban research into ectogestation, because we see the potential benefits it will bring. Rather we seek to contextualise any potential development in the prevailing and enduring norms about pregnancy to illuminate the concerns that should be considered before ectogestation is used in humans. While this investigation is inevitably speculative, it helps highlight some of the contemporary concerns about harmful conceptualisations of maternal–fetal conflict.

### Pathologising gestation

Limon notes that liberal feminists often adopt pathological language in explaining the necessity or desirability of ectogestation.^83^ Firestone described pregnancy as ‘barbaric’ and childbirth as like ‘shitting a pumpkin’.^75^ Smajdor refers in detail to the pain and suffering gestation causes women and explicitly claims it is a ‘conceptual failure in medicine and social and ethical terms to address the pathological nature of gestation and childbirth’.^77^ While Kendal advocates for ectogestation as a reproductive choice (and is explicit that she does not seek to devalue natural pregnancy and childbirth), she nevertheless describes pregnancy as ‘temporary incapacitation,’ as an illness or cause of injury, and suggests it is ‘only logical for someone to actively avoid developing a physical condition that is guaranteed to cause significant, prolonged discomfort, especially if it also carries the risk, no matter how small, of sustaining some severe injury or death’.^76^ We do not disagree that pregnancy can be difficult, harmful and in some cases dangerous. It remains true that gestating and birthing can have serious, long term, even fatal, consequences for women. However, pathologising all pregnancy could exacerbate notions of maternal–fetal conflict by explicitly locating a normal pregnancy as a source of danger and providing justification for medical intervention.

This pathologisation lends itself to the way the female body has always been ‘othered.’ Earlier, we demonstrated how the female body and particularly the womb has always been considered oppositional to and defective compared with the male body, pathologised in its ability to gestate, its inability to gestate and its capacity to menstruate. These female attributes were thus seen as medical matters worthy of medical supervision and patriarchal interference. The language of pathology that has been used by some scholars in explaining why some women might opt for ectogestation unintentionally implies that the fact that females carry pregnancies (and thus potentially subject to this ‘incapacity’ at some point or multiple times in their lifespan) renders them inferior. There are parallels between historical attitudes and the imagined ‘artificial womb’ utopia. Importantly, to pathologise and medicalise is to direct to the necessity of intervention and this can have material impacts. This is evident today in the stark increase in interference in childbirth, as the female body and its capacities, Wolf and Charles explain, are treated as an ‘inherently dangerous, unpredictable process that must be controlled to remove its dangers and lack of predictability’ because ‘serious complications can arise at any moment and create an emergency’.^62^ Burrow suggests that there is an operative technological imperative in obstetrics,^84^ which increasingly encourages individual clinicians to ‘rationalise surgical [or technological] intervention to gain as much control as possible’.^85^

Furthermore, pathologising pregnancy treats all pregnancies as homogeneous. Many women enjoy being pregnant,^14^ so we must be mindful of how using language that describes pregnancy as ‘an illness,’ analogising it to a disease or referring to it as ‘temporary incapacitation’ feeds into old-fashioned claims about the inherent pathology of female biology. This is to denigrate natural pregnancy and the women who value the experiences of pregnancy and labour. Moreover, it paints the female body as a dangerous place and feeds into claims that fetuses might be safer gestating ex utero. A woman’s body is perceived as a conflict zone to be avoided in favour of ectogestation.

### Narrative of alternative

We have examined how the womb being both invisible within the pregnant body, yet increasingly visible with a wide variety of technologies has led to the conceptualising of the pregnant body as an environment in need of supervision. The visibility of the fetus has potentially increased the prevalence of conceptualising pregnancy as a conflict-zone of competing interests. The possibility of a fetus being gestated externally further increases the visibility of the fetus and could potentially impact on how a fetus in a pregnancy is conceptualised. Sander-Saudt posits that ‘conflicts between the rights of women and fetuses will be heightened greatly as a result of this technology’.^86^ The view, even sometimes expressed in the courtroom, that the fetus is ‘a fully formed child, capable of a normal life if only it could be delivered from the mother’^87^ is potentially emboldened by technology that allows us to see, control and visualise gestation in every material way. If there is an alternative space for gestation there may be an increased tendency, as this view is already prevalent to some extent, to view the pregnant woman as a ‘temporary fetal container’.^79^ These concerns reflect aspects of Aristotle’s view of the woman as the mere ‘seed bed’.^2^

The idea of there being an alternative to the pregnancy for the fetus is consistently used inappropriately in the context of gestation to control the behaviour of pregnant women.^88^ The fact that a fetus if delivered prematurely might be able to survive in neonatal intensive care at a given fixed point (usually identified as 24 weeks) is repeatedly used as justification to control a woman’s body. After this point she is not allowed to end her pregnancy unless a fetal abnormality is present, or her health is seriously threatened. The fact that the fetus could perhaps survive ex utero—though it remains unlikely until 26 weeks^89^—prevents abortion on all but serious medical grounds. Simultaneously, she is not allowed to prematurely deliver that fetus intending for it to receive neonatal intensive care unless there is medical justification.^15^ The artificial womb is frequently posited as both an alternative to abortion,^90–92^ and to pregnancy.^78 93 94^ It is inappropriate to consider ectogestation as an alternative to abortion for three principal reasons. First, because the procedure to extract a fetus for ex uterum gestation is far more invasive than the procedures of medical or surgical abortion.^78 79^ Second, because women want access to abortion care as early as possible; most care is provided before 13 weeks,^95^ and there is not yet evidence to suggest that artificial womb technology will be capable of gestating embryos since current prototype models are reliant on fetal physiology.^81^ ^16^ Finally, several scholars have highlighted that abortion is meaningful not only a right not to be pregnant, but to encompass the broader harmful social realities for women if forced to accept the consequences of unwanted pregnancy.^79 81 83^ Romani and Horn argue that it is important to reground conversation about ectogenesis in the realities of this technology and its unsuitability as an ‘alternative to abortion’ calling for scholars to consider the ramifications of neglecting to understand abortion as healthcare.^81^

It is also harmful (and likely always going to be factually inaccurate)^17^ to label ectogestation as an alternative to pregnancy. Pence^94^ and Hammond-Browning^93^ both advocate that ectogestation might be beneficial in those instances in which a pregnant woman is behaving ‘inappropriately’, for example, abusing substances. It is thought that ectogestation brings the possibility of ‘safeguarding’ fetuses and embryos without interfering with women’s rights.^96^ It is not difficult to extrapolate from this argument that there is seemingly frustration that maternal rights are seen to be interfering with the goal of protecting a fetus (clearly placing the pregnant person, even if unintentionally, second in the pecking order) and ectogestation is thus seen as a tool to ensure these interests can be superseded. What is concerning about these arguments concerning the welfare of fetuses (and/or potential ‘ecto-children’^18^ ^93^), is that they invite the potential for ‘increased control and pressure to use ectogenesis to secure the fetus’,^80^ or to encourage compliance in a multitude of different ways with medical recommendations about behaviour during pregnancy.^96^ Welin posits that, if ectogestation were to come to fruition, ‘women who choose to have a natural pregnancy [in its place] will have to face restriction on lifestyles. At least, I believe it will be very hard to argue against such restriction in order to protect the fetus…’.^96^ This kind of argumentation is maternal–fetal conflict rearing its ugly head once more and it is reminiscent of animalculism and the view of a woman as her husband’s ‘gestational carrier.’

### Situating gestation and pregnancy

Petchesky wrote of ultrasound imagery that women must be re-centred in discussions of pregnancy with attention to context; placing the fetus ‘back into the uterus, and the uterus back into the woman’s body and her body back into its social space’.^18^ In discussions of ectogestation, there is an abject failure to recognise the realities of the technology that scholars are referring to. Arguments made about moral obligations of pregnant women or about the experience of pregnancy in the event of this technology are based on unhelpful generalisations. What is most important to highlight is that in any event the capacities of the technology mean that, first and foremost, gestation takes place inside the female body. Any claims made directly about uses of or conditions following the development of the artificial womb inevitably impact on the female body and experiences of pregnancy. Even where gestation can take place partially ex utero, it is a process that originates from and remains partially unique to the female body. Placing this reality at the centre of argumentation can prevent the subjugation of the gestating body and their autonomy.

Furthermore, appropriate language must be used to describe pregnancy and gestation that is inclusive of diverse reproductive experiences that differ person to person based on social factors, lived realities and reproductive preferences. Reproductive consciousness is individual, complex and corporeal and thus is difficult to generalise.^18^ It is crucial that natural pregnancy is not denigrated in discussions about the potential benefits of the technology. While describing the extent to which artificial womb technology can alleviate some burdens in later-term pregnancy for women who may need or choose relief, Firestone^75^ and Smajdor^77^ explicitly and Kendal^76^ implicitly use language that devalues the capacities of the female body and the empowering experiences of some pregnant women. Adopting language that is inclusive of a range of reproductive experiences can help prevent the pathologisation of gestation and assist in the conceptual understanding that the artificial womb is not a ‘direct’ alternative to a natural pregnancy that can be used to dictate the conditions of pregnancy and the behaviours of pregnant women.

Artificial wombs might be thought of, for some women, as an alternative to continuing their pregnancy at some risk to their life or health. However, the artificial womb ought not to be discussed as an ‘alternative’ in general terms to either abortion (because this claim is false^81^) or gestation. Gestation is the process of genesis of a human entity in the womb; pregnancy is the task performed by the womb and female body in sustaining gestation. An ‘artificial womb’ may be an alternative form of the process of gestation, but it is not an alternative womb (organ of the female body) or pregnancy.

Petchesky also contends that we must ‘separate the power relations within which reproductive technologies, including ultrasound imaging, are applied from the technologies themselves. If women were truly empowered in the clinic setting, as practitioners and patients, would we discard the technologies?’^18^ It is clear that ectogestation has the potential to be an incredible tool to assist pregnant women and potential parent(s) where used as an alternative to neonatal intensive care^97^ and in the absence of the concerning power dynamics outlined should be welcomed. Our task then is to mediate how such technology can come to fruition without exacerbating problematic notions of pregnancy and fetal welfare as oppositional to pregnant women; this is best done by demanding that the maternal–fetal framework is abandoned.

## Conclusion

Examining historical medical and social attitudes to women, and particularly pregnant women, helps us understand how and why misogynist tropes and damaging narratives about maternal–fetal conflict endure over time, influencing the (mis)treatment of pregnant women now and potentially in the future. We explored how historical narratives of the woman’s purpose as ‘gestational carrier’ have persisted as increasing access to the womb has influenced the perception of the fetus and its status as a potential ‘second patient.’ Historical suspicion of the womb when obscured from view has equally endured, despite increasing visibility resulting from technologies routinely used in obstetric care, as the womb, pregnancy and childbirth have institutionally been rendered an ‘emergency’^62^ warranting medical intervention. We must be mindful of these trends when speculating about future technologies and in order to minimise notions of conflict compromising care today.

It is frequently posited that a wide variety of technologies, from fetal heart rate monitoring in childbirth to ultrasound, have enabled more intervention in pregnancy.^62^ This has strengthened the perception that the fetus has distinct interests that are directly impacted on by the pregnant woman’s behaviour, which is perceived as a potential threat to those interests. We demonstrated that this conception of conflict is erroneous in several ways, both conceptually and factually. It is additionally problematic in that it fails to encompass the social context of pregnancy and the maternal–fetal unit. As Bowden explains, by ‘focussing on the behaviour of pregnant women other more significant causes of prenatal harm such as poverty and poor prenatal care are obscured and overlooked’.^30^ In order to ensure we respect women’s reproductive autonomy in a meaningful way, especially in view of a future that may bring even more innovative technologies and possibilities for intervention in pregnancy, we must abandon this overly simplistic and biased concept.

Future reproductive technologies may have emancipatory potential for women, but they may equally end up entrenching problematic patriarchal notions and gender roles. Jackson warns that advocating for ectogestation as a safer alternative for fetuses would be extremely harmful ‘since it carries the implication that the maternal body is a source of danger for the developing fetus when this is of course very seldom the case’.^79^ The possibility of advocating for ectogestation in place of pregnancy demonstrates how the artificial womb might be preferred in order to exert control over the process of gestation. The evident enthusiasm for the idea that the power of creation would no longer be contained exclusively in the female body, reveals the power of the maternal–fetal conflict narrative. We can see this in the multitude of authors who have made confident claims about a man’s entitlement to equal control over ex utero gestation.^96 98^ These seemingly echo the historical calls of medical men and putative fathers in their attempts to assert control over reproduction. We; therefore, should be mindful of these concerns in the development of technology that, in attempting to emulate gestation, has promising benefits for the care of preterm neonates and for women experiencing dangerous pregnancies. Reorienting our understanding of pregnancy away from maternal–fetal conflict will ensure that potential benefits from future assistive technologies like ectogestation can be realised, but also will benefit pregnant women experiencing problems resulting from conflict in contemporary prenatal care.

## Footnotes

It is important to acknowledge that it is persons of female biology, regardless of the gender they live in or identify as, that can become pregnant. In this article, we refer to women and pregnant women because throughout history the fact that the majority of pregnant people identified as, or were assumed to be, women because of their biology has impacted on how pregnancy has been and is conceptualised and how pregnant people were and are treated.

In December 2014 the first baby was born from a transplanted uterus in Sweden.^99^

In 2017 a team of fetal scientists and surgeons in Philadelphia revealed an AW prototype that had yielded promising results in animal testing (the Biobag). Another research team, based in Western Australia/Japan, has reported similar results from testing their prototype AW, the EVE platform. In 2019 a third research team in the Netherlands announced they had received Horizon 2020 funding to build their AW prototype.^71–73^

Medical objections to uterus transplantation in non-biologically female persons (that formed the basis of the Montreal Criteria about ethical uterus transplantation) do not appear to be well-founded.^100^

Claims that artificial wombs will de-gender gestation are frequently made in the literature.^96 98 101 102^ For an explanation of why these claims are inaccurate see.^103^

Kingma and Finn note that it is more appropriate to refer to ectogestation and the specific names of designed prototypes because describing the technologies as ‘artificial wombs’ is inaccurate,^58^ and as we will demonstrate the notion of an ‘artificial’ womb might perpetuate harmful narratives about the need to control gestation. Here, we use the term ‘artificial womb’ because this is how they are popularly referred to and understood, and because this term is an important part of our argument about how language is used in this context to create narratives about women.

The right to sexual intercourse was not solely linked to the right to reproduce but theological suspicions of sexual pleasure even in marriage, the notion that the primary purpose of marriage was the procreation of children in theory demoted the non-procreative role of marital intercourse to a subordinate role.

For example: Alabama, Georgia, Kentucky, Louisiana, Mississippi, Missouri and Ohio.

This is also the case in several other common-law jurisdictions; the Born-Alive rule is enshrined in the Canadian Criminal Code, for example.

There are compelling calls to decriminalise abortion in England and Wales in order to afford proper weight to the bodily autonomy of pregnant women.^51^

This was also confirmed in the European Court of Human Rights Decision in *Vo v France*.^104^

*Re MB* 53 left the door open to problematic findings of incapacity. In finding that panic and a phobia of needles incapacitated a pregnant woman, this judgment left open the possibility of using terms like ‘panic’ and ‘pain’ generally to establish a person is incapable of making decisions. Since panic and pain are very common, normal and temporary states they might easily be attributed to a pregnant woman and used to conclude she does not have capacity by virtue of the fact she is in childbirth. This may ‘tempt’ a concerned judge to ‘err on the side of finding incompetence’ especially when pregnant women are in disagreement with their doctors, but pain prevents clear, reasoned explanation.^54^ There are similar concerns about forced caesareans in the United States.^67^

Even if not legally recognised as a person the fetus does have some legally protected interests, for example in the Abortion Act.^51 66 105^

There are many women (and non-women) campaigning for a right to gestate. For example, those who want to receive a womb transplant in order to be able to carry a pregnancy or women who campaign for access to IVF treatment.

For example, her life is threatened by a condition like preeclampsia or the fetus is displaying signs of intrauterine growth restriction.

Model AW prototypes currently being tested on animals are reliant on the subject being developed beyond an embryo; for example, it must have a primitive heartbeat to enable circulation.^71 97^

It is hard to imagine a technology that could emulate natural pregnancy so well that it was literally a direct alternative to pregnancy.

Inevitably referring to the subject of an artificial womb as an ‘ecto-child’^93^ uses emotive language to describe the entity that can be potentially used to compel behaviour during pregnancy. This is one of the reasons why the term ‘gestateling’ [105] for the subject of the artificial womb is thought to be important.
